# *In vitro *and *in vivo *plasmalogen replacement evaluations in rhizomelic chrondrodysplasia punctata and Pelizaeus-Merzbacher disease using PPI-1011, an ether lipid plasmalogen precursor

**DOI:** 10.1186/1476-511X-10-182

**Published:** 2011-10-18

**Authors:** Paul L Wood, M Amin Khan, Tara Smith, Greg Ehrmantraut, Wei Jin, Wei Cui, Nancy E Braverman, Dayan B Goodenowe

**Affiliations:** 1 Dept. of Pharmacology, DeBusk College of Osteopathic Medicine, Lincoln Memorial University, 6965 Cumberland Gap Parkway, Harrogate, TN, 37752, USA; 2 R&D Dept., Phenomenome Discoveries Inc, 204-407 Downey Road, Saskatoon, SK, S7N 4L8, Canada; 3 Depts. of Human Genetics and Pediatrics, McGill University-Montreal Children's Hospital Research Institute, 4060 Ste-Catherine West, PT-406.2, Montreal, QC, H3Z 2Z3, Canada

**Keywords:** Rhizomelic chrondrodysplasia punctata type 1, Rhizomelic chrondrodysplasia punctata type 2, Pelizaeus-Merzbacher disease, Pex7 mouse, lymphocytes, plasmalogen precursor, DHA, peroxisomal disorders, PPI-1011

## Abstract

**Background:**

Childhood peroxisomal disorders and leukodystrophies are devastating diseases characterized by dysfunctional lipid metabolism. Plasmalogens (ether glycerophosphoethanolamine lipids) are decreased in these genetic disorders. The biosynthesis of plasmalogens is initiated in peroxisomes but completed in the endoplasmic reticulum. We therefore undertook a study to evaluate the ability of a 3-substituted, 1-alkyl, 2-acyl glyceryl ether lipid (PPI-1011) to replace plasmalogens in rhizomelic chrondrodysplasia punctata type 1 (RCDP1) and rhizomelic chrondrodysplasia punctata type 2 (RCDP2) lymphocytes which possess peroxisomal mutations culminating in deficient plasmalogen synthesis. We also examined plasmalogen synthesis in Pelizaeus-Merzbacher disease (PMD) lymphocytes which possess a proteolipid protein-1 (PLP1) missense mutation that results in abnormal PLP1 folding and it's accumulation in the endoplasmic reticulum (ER), the cellular site of the last steps in plasmalogen synthesis. *In vivo *incorporation of plasmalogen precursor into tissue plasmalogens was also evaluated in the Pex7 mouse model of plasmalogen deficiency.

**Results:**

In both RCDP1 and RCDP2 lymphocytes, PPI-1011 repleted the target ethanolamine plasmalogen (PlsEtn16:0/22:6) in a concentration dependent manner. In addition, deacylation/reacylation reactions resulted in repletion of PlsEtn 16:0/20:4 in both RCDP1 and RCDP2 lymphocytes, repletion of PlsEtn 16:0/18:1 and PlsEtn 16:0/18:2 in RCDP2 lymphocytes, and partial repletion of PlsEtn 16:0/18:1 and PlsEtn 16:0/18:2 in RCDP1 lymphocytes. In the Pex7 mouse, oral dosing of labeled PPI-1011 demonstrated repletion of tissue levels of the target plasmalogen PlsEtn 16:0/22:6 with phospholipid remodeling also resulting in significant repletion of PlsEtn 16:0/20:4 and PlsEtn 16:0/18:1. Metabolic conversion of PPI-1011 to the target plasmalogen was most active in the liver.

**Conclusions:**

Our data demonstrate that PPI-1011 is activated (removal of 3-substitution) and converted to PlsEtn *in vitro *in both RCDP1 and RCDP2 lymphocytes and *in vivo *in the Pex7 mouse model of RCPD1 effectively bypassing the peroxisomal dysfunction present in these disorders. While PPI-1011 was shown to replete PlsEtns 16:0/x, ether lipid precursors of PlsEtn 18:0/x and PlsEtn 18:1/x may also be needed to achieve optimal clinical benefits of plasmalogen replacement in these complex patient populations. In contrast, only limited plasmalogen replacement was observed in PMD lymphocytes suggesting that the effects of protein misfolding and accumulation in the ER negatively affect processing of plasmalogen precursors in this cellular compartment.

## Background

The peroxisome disorder, rhizomelic chrondrodysplasia punctata (RCDP) is a devasting disease characterized by severe growth retardation and developmental delays. Most children do not survive beyond 10 years of age and death is often secondary to respiratory illnesses [1]. The clinical features of RCDP are a direct result of plasmalogen deficiency. Two peroxisomal enzymes, acyl CoA:dihydroxyacetonephosphate acyltransferase (GNPAT; EC 2.3.1.42) and alkyl-dihydroxyacetone phosphate synthase (AGPS; EC 2.5.1.26), are critical for the committing steps of ether lipid plasmalogen synthesis [2]. RCDP is a heterogeneous autosomal recessive disorder [3-5] most commonly caused by defects in the PEX7, the peroxisome transporter for AGPS (RCDP1), but also by defects in the enzymes themselves, GNPAT, (RCDP2) or AGPS (RCDP3). After synthesis of alkylglycerol precursors in the peroxisome, the synthesis of ether phospholipids are completed in the ER. Nevertheless, the only known inherited defects in plasmalogen synthesis are the peroxisomal defects.

Recently, we performed a lipidomics analysis [6] in Pelizaeus-Merzbacher disease (PMD) fibroblasts and lymphocytes [7-9], in which we demonstrated significant reduction in plasmalogen levels. However the etiology for this plasmalogen deficiency is unknown.

Since there are no treatments for these disorders, we undertook an evaluation of the ability of PPI-1011, a DHA-containing ether lipid plasmalogen precursor which bypasses the requirement for peroxisomes, to augment deficient cellular plasmalogens in RCDP1, RCDP2 and PMD lymphocytes and in the murine Pex7 model of RCDP1 [10].

## Materials and methods

### Cell Culture

Control lymphocytes (Coriell GM13072 and GM02184); PMD lymphocytes (Coriell GM09545; PLP1 c.767C > T or p.P215S); RCDP1 lymphocytes (Coriell GM09291; PEX7 c.870 871insCAA/875T > A or p.C290 E291insQ/L292X and show a severe plasmalogen synthesis defect) and RCDP2 lymphocytes (Coriell GM16776; homozygous for GNPT c.1280-3T > G predicted to encode an in-frame protein p.427 507 del and showing a milder plasmalogen synthesis defect) [11] were kept as suspension cultures (25 ml flasks) in RPMI 1640 (Hyclone) supplemented with 10% FBS and 1% antibiotic/antimycotic, at 37°C in a 5% CO_2 _incubator [6].

Lymphocytes were harvested (1280 ×g) and washed twice with cold phosphate buffered saline (PBS) and the stored at -80°C for subsequent lipidomic analyses.

### Plasmalogen Synthesis

To monitor plasmalogen synthesis lymphocytes were incubated with 20 or 100 uM PPI-1011 [7,12] or PPI-1038 for 72 hr and incorporation into cellular plasmalogens measured. PPI-1038 is a stable isotopic version of PPI-1011, an ether lipid plasmalogen precursor with a [^13^C_3_]glycerol backbone (G), a [^13^C_16_]palmitic ether linkage (P) at sn-1, a [^13^C_3_]DHA (D) acyl linkage at sn-2, and a lipoic acid acyl linkage at sn-3 to stabilize the precursor. Incorporation into the target plasmalogen was monitored as [^13^C_22_]16:0/22:6 PlsEtn (P-G-D). Lipid remodeling, which involves deacylation at sn-2 (i.e. removal of [^13^C_3_]DHA) and reacylation with other fatty acids was monitored as [^13^C_19_]16:0/x PlsEtns (P-G). Acylation of unlabelled plasmalogen precursors with the released [^13^C_3_]DHA was monitored as [^13^C_3_]x/22:6 PlsEtns (D). The specificity of these measurements was achieved via specific LC-MS/MS MRMs.

### Pex7 Mice

Levels of plasmalogens in different tissues were measured in Pex7 (18 to 24 g) mice [10] and both heterozygote and wild-type controls (24-30 g). No differences in plasmalogen levels were noted between wild-type and heterozygote controls. In the subsequent experiment, Pex7 mice and heterozygote controls were orally dosed by gavage with PPI-1038 (100 mg/kg; 10 mg/ml Neobee M5; Spectrum Chemical Mfg.) daily for 3 days. On day 4, tissues were harvested for plasmalogen analysis. All mice were studied between ages 2 and 3 months. The mouse studies were conducted under the McGill University Animal Care Committee approved protocol 5538 entitled "Study of PEX7 deficient mice as models for RCDP''.

### Plasmalogen Analyses

For plasmalogen analyses, cells or pulverized tissues were sonicated in 1 mL of PBS + 0.5 mL methanol. Next, 2 mL tert-butylmethylether were added and the samples capped and shaken (1400 rpm) for 10 min at room temperature. The samples were then centrifuged for 8 min in a clinical centrifuge and 1 ml of the upper organic layer isolated for LC-MS/MS analyses of endogenous and labeled ethanolamine plasmalogens as reported previously [7,12-14].

### Data Analyses

*In vitro *data are presented as mean ± SEM for groups of six to eight 25 ml flasks. Since standards are not available for the lipidomic analysis of diverse plasmalogens, these were normalized to the housekeeping metabolite PtdEtn 16:0/18:0. Data were analyzed by 1-way ANOVA, followed by the Tukey-Kramer test to determine differences between groups.

*In vivo *data are presented as mean ± SEM for groups of 6 for plasmalogen levels and as mean ± SD for groups of 4 mice for the precursor labeling study. HO comparisons to HT mice were conducted with a t-test.

## Results

### Ethanolamine Plasmalogens in RCDP Lymphocytes

**Figure 1:**
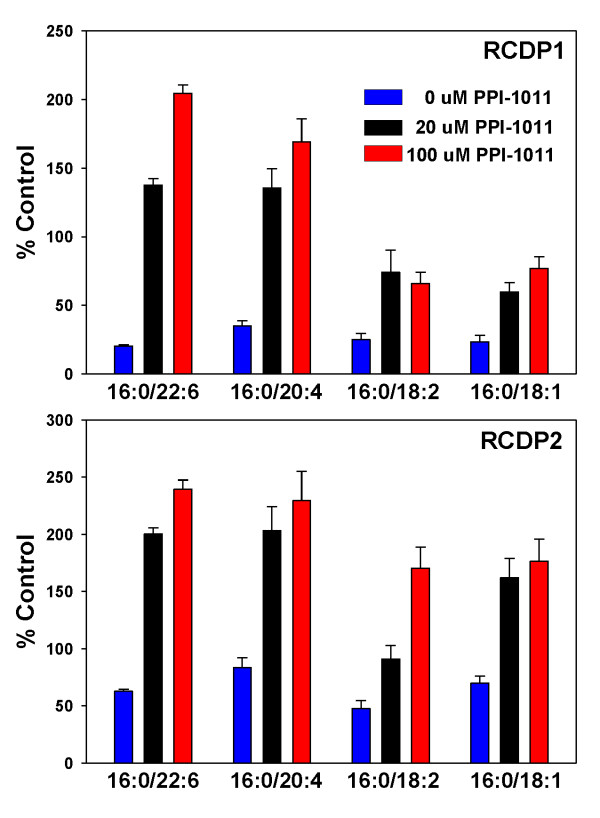
**Ethanolamine plasmalogen levels in RCDP1 and RCDP2 lymphocytes with 0, 20 or 100 μM PPI-1011 for 72 hr**. N = 8. Mean ± SEM. All basal decrements and PPI-1011-dependent increases were significantly (p < 0.01) from control lymphocytes and from 0 μM PPI-1011, respectively. 16:0 (palmitic acid), 18:1 (oleic acid), 18:2 (linoleic acid), 20:4 (arachidonic acid), 22:6 (docosahexaenoic acid; DHA).

In RCDP1 lymphocytes, PlsEtns 16:0/x were all decreased to approximately 25% of control (Figure 1). The ether lipid plasmalogen precursor, PPI-1011 effectively restored PlsEtn 16:0/22:6 and PlsEtn 16:0/22:4 and partially restored PlsEtn 16:0/18:2 and PlsEtn 16:0/18:1 (Figure 1).

In RCDP2 lymphocytes PlsEtns 16:0/x were all decreased to approximately 60% of control (Figure 1). All plasmalogens were augmented beyond control levels with PPI-1011 treatment (Figure 1).

In both RCDP1 and RCDP2 lymphocytes, PPI-1011 did not augment PlsEtns 18:0/x or PlsEtns 18:1/x (data not shown).

### Ethanolamine Plasmalogen Synthesis in RCDP and PMD Lymphocytes

**Figure 2:**
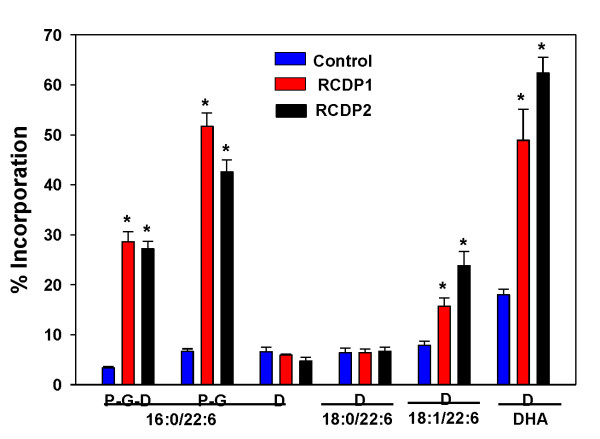
**Incorporation of PPI-1038 (100 μM; 72 hr) into the 16:0/22:6, 18:0/22:6 and 18:1/22:6 plasmalogens and DHA of RCDP1 and RCDP2 lymphocytes**. N = 6. Mean ± SEM. P =[^13^C_16_]palmitic acid; G =[^13^C_3_]glycerol; D = [^13^C_3_]DHA. *, p < 0.01 vs. control.

Incorporation of intact labeled (P-G-D = [^13^C_16_]Palmitate-[^13^C_3_]Glycerol-[^13^C_3_]-DHA) and remodeled (P-G) PPI-1038, into the target plasmalogen (PlsEtn 16:0/22:6) was significantly increased in both RCDP1 and RCDP2 lymphocytes (Figure 2). The labeled DHA pool formed by deacylation at sn-2 was significantly increased in both RCDP1 and RCDP2 lymphocytes (Figure 2). However, increased utilization of this DHA pool for reacylation at sn-2 was only observed in PlsEtn 18:1/22:6 (Figure 2).

**Figure 3:**
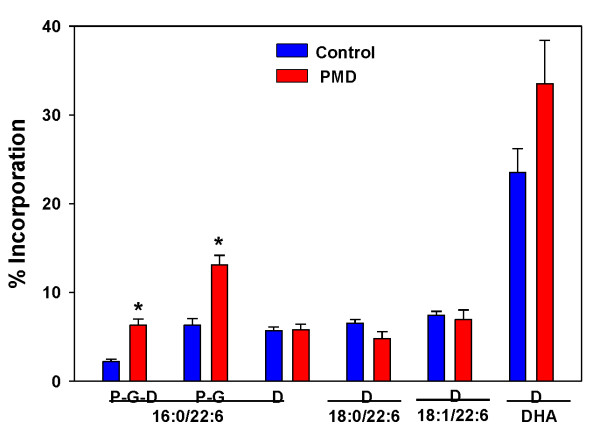
**Incorporation of PPI-1038 (100 μM; 72 hr) into the 16:0/22:6, 18:0/22:6 and 18:1/22:6 plasmalogens and DHA of PMD lymphocytes**. N = 6. Mean ± SEM. P =[^13^C_6_]palmitic acid; G =[^13^C_3_]glycerol; D = [^13^C_3_]DHA. *, p < 0.01 vs. control.

Incorporation of intact labeled (P-G-D) and remodeled (P-G) PPI-1038, into the target plasmalogen (PlsEtn 16:0/22:6) was significantly increased in PMD lymphocytes (Figure 3), but much less than that observed with RCDP lymphocytes (Figure 2).

### Pex7 Mice

**Figure 4:**
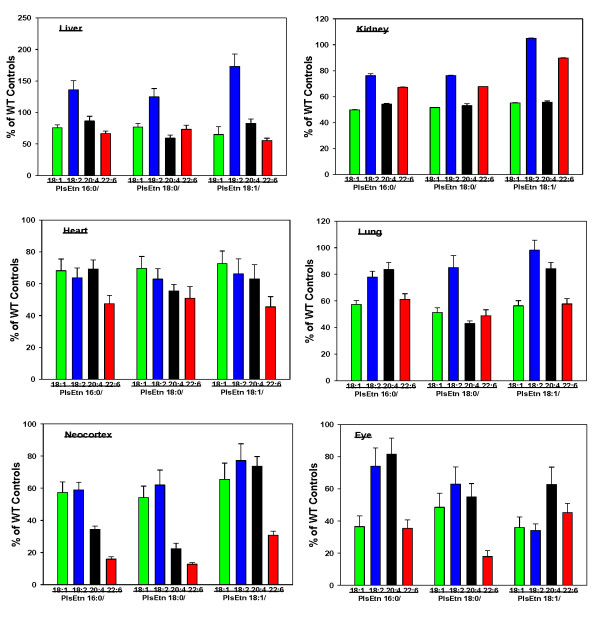
**Ethanolamine plasmalogen levels in Pex7 mouse tissues (liver, kidney, heart, lung, neocortex and eye)**. N = 5 Pex 7; N = 10 controls (2 heterozygotes + 8 wild-type). Mean ± SEM. All plasmalogen decreases were statistically significant (p < 0.05).

Tissue levels of PlsEtns 16:0/x were significantly decreased in the liver, kidneys, heart, lungs, neocortex and eyes of Pex7 mice (Figure 4). These decreases were approximately 50% except for PlsEtns 16:0/22:6 and 18:0/22:6 which were reduced to 15 to 30% of control in the eye and neocortex (Figure 4).

**Figure 5:**
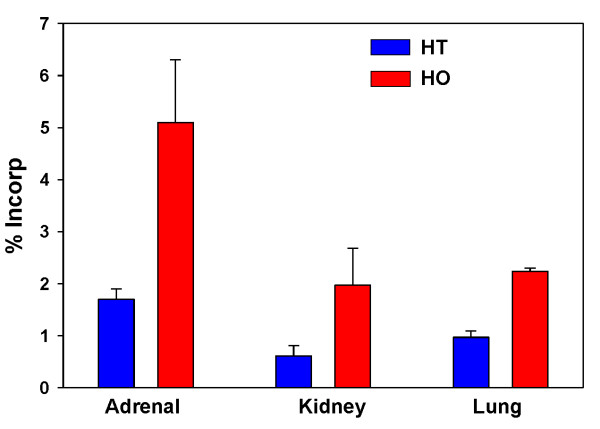
**Incorporation of [^13^C_16_]palmitic acid and [^13^C_3_]glycerol from PPI-1038 (100 mg/kg/day for 3 days) into Pex7 tissue PlsEtn 16:0/22:6**. N = 4. Mean ± SD. Increases in labeling in the Pex7 mice were significant in all cases (p < 0.01). HT, heterozygote controls; HO, homozygotes.

**Figure 6:**
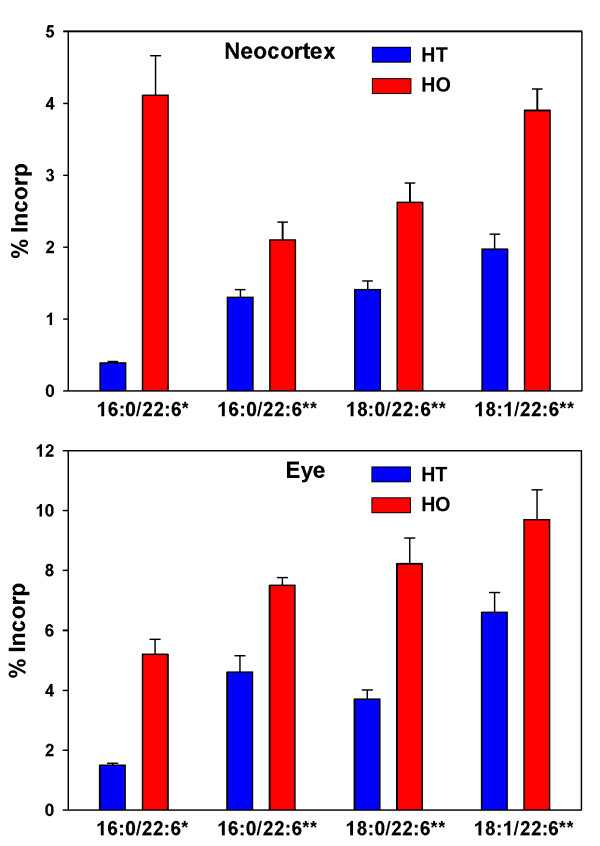
Incorporation of [^13^C_6_]palmitic acid and [^13^C_3_]glycerol (*) from PPI-1038 (100 mg/kg/day for 3 days) into PlsEtn 16:0/22:6 in the neocortex and eyes of Pex7 mice. Similarly the incorporation of [^13^C_3_]DHA (**) into PlsEtn 16:0/22:6, PlsEtn 18:0/22:6 and PlsEtn 18:1/22:6 is presented. N = 4. Mean ± SD. Increases in labeling in the Pex7 mice were significant in all cases (p < 0.01). HT, heterozygote controls; HO, homozygotes. * = [^13^C_16_]palmitic acid + [^13^C_3_]glycerol labeled PlsEtn x/22:6; ** = [^13^C_3_]DHA labeled PlsEn x/22:6.

Administration of PPI-1038, daily for 3 days, resulted in significant labeling of the PlsEtn 16:0/22:6 pool in all tissues. The major labeled form in the adrenal, kidney, lung and liver was the [^13^C_16_]palmitic acid + [^13^C_3_]glycerol labeled PlsEtn 16:0/22:6, resulting from lipid remodeling at sn-2. The data for the adrenal, kidney and lung are presented in Figure 5. Much greater labeling in the liver was observed: 24.9 ± 3.1% for heterozygote controls and 40.3 ± 4.4% for homozygote Pex7 mice. In the case of the brain (neocortex) and eyes, increased [^13^C_16_]palmitic acid + [^13^C_3_]glycerol labeled PlsEtn 16:0/22:6 was measured (Figure 6). In these tissues, the [^13^C_3_]DHA released by deacylation at sn-2 was incorporated into PlsEtn 16:0/22:6, PlsEtn 18:0/22:6 and PlsEtn 18:1/22:6 (Figure 6).

## Discussion

**Figure 7:**
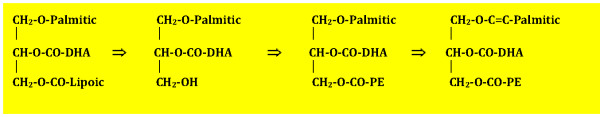
**Conversion of PPI-1011 to the target plasmalogen PlsEtn 16:0/22:6**. Removal of the sn-3 lipoic acid by lipases is followed in the endoplasmic reticulum by addition of phosphoethanolamine to the glycerol backbone at sn-3 (EC 3.1.3.4) and desaturation of the ether linked fatty acid at sn-1 (EC 1.14.99.19). Subsequent lipid remodeling at sn-2 is mediated by tightly coupled deacylation/reacylation enzyme reactions [10].

RCDP is a lethal disorder of critical peroxisomal genes involved in ether lipid synthesis, particularly ethanolamine plasmalogens (PlsEtn). Strategies to replace plasmalogens must take these deficiencies into account and supply ether lipid precursors that are capable of bypassing abnormal peroxisomal function. Our data demonstrate that PPI-1011 can bypass the requirements for functional peroxisomes since PPI-1011 efficiently replaced the target PlsEtn 16:0/22:6 (Figure 7) in both RCDP1 and RCDP2 lymphocytes. In addition, this target plasmalogen underwent significant lipid remodeling at sn-2 to also replenish other PlsEtns 16:0/x. No augmentation of PlsEtns 18:0/x or PlsEtns 18:1/x were detected, suggesting that a combination of 16:0, 18:0 and 18:1 ether lipid precursors may be needed to obtain the best potential clinical outcome for plasmalogen precursors in RCDP clinical trials.

The Pex7 hypomorphic mouse has been shown to possess approximately 50% reductions in plasmalogens, assayed by a procedure that does not distinguish the multiple plasmalogen species [9]. Our LC-MS/MS analyses also demonstrated an approximate 50% decrease in cellular pools of plasmalogens but also detected much more dramatic decrements in DHA-containing plasmalogens in the eye and brain. These tissues are highly dependent upon DHA and DHA-containing plasmalogens [15,16] and possess specific transport mechanisms to import plasmalogens and plasmalogen precursors [17,18]. Our studies with labeled PPI-1011 (PPI-1038) demonstrated that this ether lipid precursor is orally bioavailable and generates the target plasmalogen (PlsEtn 16:0/22:6; Figure 6) in a number of control mouse tissues. Our data further emphasize the importance of the liver in the synthesis of critical peroxisome-dependent CNS plasmalogens, as previously shown for DHA [16]. In addition this plasmalogen synthesis from labeled PPI-1011 is augmented in Pex7 deficient mice. The lack of lipid remodeling at sn-1 again indicates that a cocktail of 16:0, 18:0 and 18:1 ether lipid precursors to obtain a therapeutic effect in RCDP may be needed.

Ethanolamine plasmalogen synthesis is complicated in that multiple cellular compartments are involved [19]. In the case of PMD, the combination of aberrant function of peroxisomes and the endoplasmic reticulum [20,21] results in decrements in plasmalogens [7]. Our data with PMD lymphocytes demonstrate that this combination of cellular defects limits the ability of ether lipid precursors to resupply plasmalogens and that this is unlikely to be a fruitful therapeutic approach for PMD.

In summary, these data demonstrate that ether lipid precursors can bypass dysfunctional peroxisomes and replace critical plasmalogens in RCDP lymphocytes and in the Pex7 mouse model of RCDP1. These data also indicate that early and sustained supply of a combination of 16:0, 18:0 and 18:1 ether lipid precursors may be the optimal translational path for a clinical study. This is a hypothesis that we will first validate in the Pex7 mouse. Our data also suggest that plasmalogen replacement in PMD with ether lipid precursors is unlikely to be a viable strategy.

## List of abbreviations

16:0: palmitic acid; 18:0: stearic acid; 18:1: oleic acid; 18:2: linoleic acid; 20:4: arachidonic acid; 22:6: docosahexaenoic acid (DHA); HO: homozygote; HT: heterozygote; PlsEtn: ethanolamine plasmalogen; PMD: Pelizaeus-Merzbacher disease; RCDP: rhizomelic chrondrodysplasia punctata.

## Competing interests

PW, AK, GE, TS and DG are all involved in the preclinical development of PPI-1011.

## Authors' contributions

All authors read and approved the manuscript. All authors participated in the study design, supervision of assay QA/QC and data interpretation. TS, GE, WJ, WC and PW performed experiments.
